# Supplementary material for: A dysmorphic mouse model reveals developmental interactions of chondrocranium and dermatocranium
Source: eLife. 2022 Jun 15;11:e76653. doi: 10.7554/eLife.76653 (PMC9259032; doi:10.7554/eLife.76653)
Supplement: Transparent reporting form [file elife-76653-transrepform1.docx]

***eLife’s* transparent reporting form**

We encourage authors to provide detailed information *within their submission* to facilitate the interpretation and replication of experiments. Authors can upload supporting documentation to indicate the use of appropriate reporting guidelines for health-related research (see [EQUATOR Network](http://www.equator-network.org/)), life science research (see the [BioSharing Information Resource](https://biosharing.org/)), or the [ARRIVE guidelines](http://www.plosbiology.org/article/info:doi/10.1371/journal.pbio.1000412) for reporting work involving animal research. Where applicable, authors should refer to any relevant reporting standards documents in this form.

If you have any questions, please consult our Journal Policies and/or contact us: [editorial@elifesciences.org](mailto:editorial@elifesciences.org).

**Sample-size estimation**

- You should state whether an appropriate sample size was computed when the study was being designed
- You should state the statistical method of sample size computation and any required assumptions
- If no explicit power analysis was used, you should describe how you decided what sample (replicate) size (number) to use

Please outline where this information can be found within the submission (e.g., sections or figure legends), or explain why this information doesn’t apply to your submission:

Sample sizes are found in the Materials and Methods section in Table 6. To determine the minimum number of animals/group to ensure adequate statistical power of µCT data, we applied a two-sided Student's t-test to linear measures collected from 3D reconstructions of µCT images of mouse affected and unaffected mouse embryos. Given the experimental design: two-sided, unpaired (cases and controls independent) and assuming type I error probability = 0.05, 4 to 6 animals/group were needed. At least three images were measured per region per individual (See Table 6 for n) for histological studies.

**Replicates**

- You should report how often each experiment was performed
- You should include a definition of biological versus technical replication
- The data obtained should be provided and sufficient information should be provided to indicate the number of independent biological and/or technical replicates
- If you encountered any outliers, you should describe how these were handled
- Criteria for exclusion/inclusion of data should be clearly stated
- High-throughput sequence data should be uploaded before submission, with a private link for reviewers provided (these are available from both GEO and ArrayExpress)

Please outline where this information can be found within the submission (e.g., sections or figure legends), or explain why this information doesn’t apply to your submission:

For µCT analysis, each specimen in Table 6 underwent CT imaging and was included based upon quality of images, primarily based upon adequate contrast between specific tissues of interest. Specimens with obvious cleft palate were excluded from µCT and all other analyses. As described the Data Collection section, a hydroxy apatite (HA) phantom was included alongside specimens being scanned for bone, and a minimum of 70-100 mg/cm^3^ partial density HA was used to reconstruct bony surfaces for all ages/genotypes. Phosphotungstic acid-enhanced (PTA) scans were processed using an automatic deep learning based fully convoluted networks as described in the subsection “Segmentation of embryonic cartilage”. Manual annotation performed by experts provided very sparse annotation for automatic chondrocranium segmentation via uncertainty guided self-training. The model was then fine-tuned via human-aided corrections of specific regions of interest. We evaluated inadequately segmented regions and manually corrected the algorithm-generated predictions, and then combined the annotations thus obtained and pseudo-labels to further fine-tune the segmentation model. Consequently, most specimens were segmented adequately by our model, except for extremely thin, small, or ambiguous regions in certain specimens. Finally, we manually corrected these local errors to generate an accurate chondrocranium model for quantitative analysis. The isosurfaces for bone and chondrocranium of each specimen were then digitized twice by the same observer, checked for gross error, and measurement error was minimized by averaging the coordinates of the two trials. A maximum of 5% error in landmark placement was accepted. At least three images were measured per region per individual for histological studies (See Table 6 for sample sizes). At least three images were measured per region per individual for histological studies.

**Statistical reporting**

- Statistical analysis methods should be described and justified
- Raw data should be presented in figures whenever informative to do so (typically when N per group is less than 10)
- For each experiment, you should identify the statistical tests used, exact values of N, definitions of center, methods of multiple test correction, and dispersion and precision measures (e.g., mean, median, SD, SEM, confidence intervals; and, for the major substantive results, a measure of effect size (e.g., Pearson's r, Cohen's d)
- Report exact p-values wherever possible alongside the summary statistics and 95% confidence intervals. These should be reported for all key questions and not only when the p-value is less than 0.05.

Please outline where this information can be found within the submission (e.g., sections or figure legends), or explain why this information doesn’t apply to your submission:

The Statistical Analyses section describes methods. Euclidean Distance Matrix Analysis (EDMA) was used to test for morphological differences between *Fgfr2c^C342Y/+^* and *Fgfr2c^+/+^* mice using a boot-strapped hypothesis testing procedure and a non-parametric bootstrapping procedure was used to estimate 90% confidence intervals for all unique linear distances estimated between landmarks. Ontogenetic variation in chondrocranial and skull shapes were assessed using principal components analysis (PCA) of the complete set of linear distances estimated among landmarks. We statistically compared patterns of morphological integration (MI) of 3D landmark data using a bootstrap-based method implemented in MIBoot software. If the correlation matrices are the same for two samples, then the correlation-difference matrix consists of zeros. If they are not similar, each element of the correlation difference matrix (representing the difference between a single linear distance) is statistically evaluated using a nonparametric bootstrap approach to estimate confidence intervals (α = 0.10). If a confidence interval does not include zero (the expected value under the null hypothesis of similarity), then the null hypothesis of similar associations for that linear distance pair is rejected. Non-parametric Mann-Whitney U tests were used to compare bone volumes, histological, and histomorphometric measures of genotypes at each age in SPSS 25 software, as there were violations of assumptions of homogeneity of variance and/or normality.

(For large datasets, or papers with a very large number of statistical tests, you may upload a single table file with tests, Ns, etc., with reference to sections in the manuscript.)

**Group allocation**

- Indicate how samples were allocated into experimental groups (in the case of clinical studies, please specify allocation to treatment method); if randomization was used, please also state if restricted randomization was applied
- Indicate if masking was used during group allocation, data collection and/or data analysis

Please outline where this information can be found within the submission (e.g., sections or figure legends), or explain why this information doesn’t apply to your submission:

Groups were determined based upon age (according to conception date) and genotype (by PCR) throughout all experiments as indicated in Table 6. There were no morphological differences between sexes in embryonic specimens, so males and females were combined. Researchers were masked to the genotype of specimens for data collection and analysis when possible. Randomly selected specimens per age and genotype were labeled to conceal genotype for histological processing.

**Additional data files (“source data”)**

- We encourage you to upload relevant additional data files, such as numerical data that are represented as a graph in a figure, or as a summary table
- Where provided, these should be in the most useful format, and they can be uploaded as “Source data” files linked to a main figure or table
- Include model definition files including the full list of parameters used
- Include code used for data analysis (e.g., R, MatLab)
- Avoid stating that data files are “available upon request”

Please indicate the figures or tables for which source data files have been provided:

Due to the large size of the majority of these data, data have been made available through Penn State University Libraries ScholarSphere repository at DOI 10.26207/qgke-r185 and include: bone micro-CT images, PTA-e micro-CT images, 3D reconstruction examples of the chondrocrania of one unaffected (*Fgfr2c^+/+^)* and one affected (*Fgfr2c^C342Y/+^*) at E13.5, E14.5, E15.5, E16.5, and E17.5, bone volumes, histological images, histomorphometric data, 3D landmark coordinate data, correlation matrices estimated by MIboot used in MI analyses, PCA output, and suture scores. Information on how to download the WinEDMA programs can be found at <https://getahead.la.psu.edu/resources/edma> and the EDMAinR programs are available on github (https://github.com/psolymos/EDMAinR). Code for automatic chondrocranium segmentation with very sparse annotation via uncertainty-guided self-training is available through <https://github.com/ndcse-medical/CartSeg_UGST>. PTA-e staining protocols for various embryonic ages of mice are available: <https://doi.org/10.1002/dvdy.136>
